# Supplementary material for: Multi-Time-Point Fecal Sampling in Human and Mouse Reveals the Formation of New Homeostasis in Gut Microbiota after Bowel Cleansing
Source: Microorganisms. 2022 Nov 23;10(12):2317. doi: 10.3390/microorganisms10122317 (PMC9783159; doi:10.3390/microorganisms10122317)
Supplement: Supplementary file 1 [file microorganisms-10-02317-s001.zip › microorganisms-2014119-supplementary.pdf]

Supplementary Information

Table S1: Significant microbial changes between before and after bowel cleansing

Table S2: Comparison of microbiota changes between humans and mice after bowel cleansing

Figure S1: The alpha diversity of the microbiota after bowel cleansing over time

Figure S2: Evaluation of the humanization effect on mice

Figure S3: LEfSe analysis was performed on the key bacteria that changed after colon cleansing

Table S1 Significant microbial changes between before and after bowel cleansing

Phylum	Genus	Before (%)	After (%)
Actinobacteria	*Bifidobacterium*	1.55	1.78*
Bacteroidetes	*Bacteroides*	27.93	40.92*
Firmicutes	*Roseburia*	1.92	0.50*
	Eubacterium	1.14	0.21*
	Blautia	8.36	2.24
	Ruminococcus gauvreauii	0.52	0.05
	Agathobacter	1.32	0.14
	Fusicatenibacter	1.58	0.36
	Dorea	1.07	0.39
	Lactobacillus	0.21	0.05

The data represent the relative abundance of the microbiota. These are the bacteria that showed a trend of change after Wilicoxon paired signed-rank test; Before, before bowel cleansing; After, first sampling after bowel cleansing; *Statistically significantly different from sample before bowel cleansing, $p<0.05$.

Table S2 Comparison of microbiota changes between humans and mice after bowel cleansing

Phylum	Genus	Human (%)	Mice (%)
Actinobacteria	*Bifidobacterium*	-3.32	-0.06
Bacteroidetes	*Bacteroides*	+13.00	+12.27
Firmicutes	*Roseburia*	-1.42	-0.39
	Eubacterium	-0.92	-0.42
	Blautia	-6.12	+0.16
	Ruminococcus gauvreauii	-0.46	-0.48
	Agathobacter	-1.17	\
	Fusicatenibacter	-1.23	-0.06
	Dorea	-0.68	0
	Lactobacillus	-0.16	-11.26

The data represent the relative abundance change of the microbiota in human and mice before and after bowel cleansing.

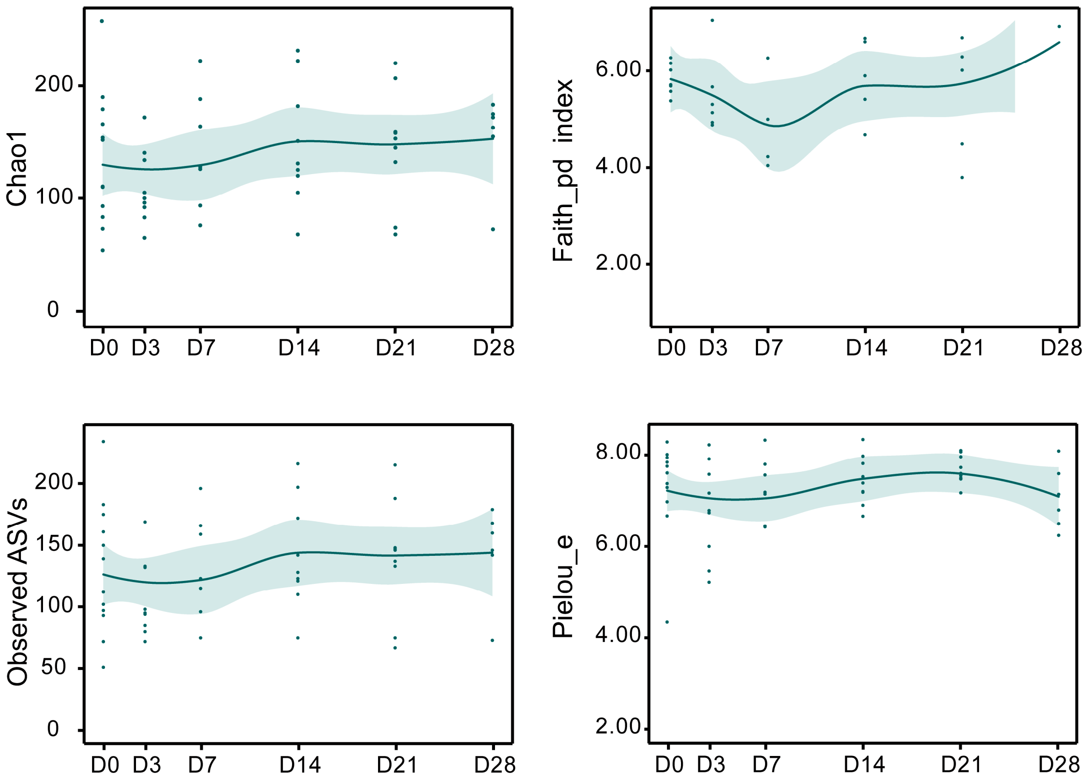

Figure S1 The alpha diversity of the microbiota after bowel preparation over time.

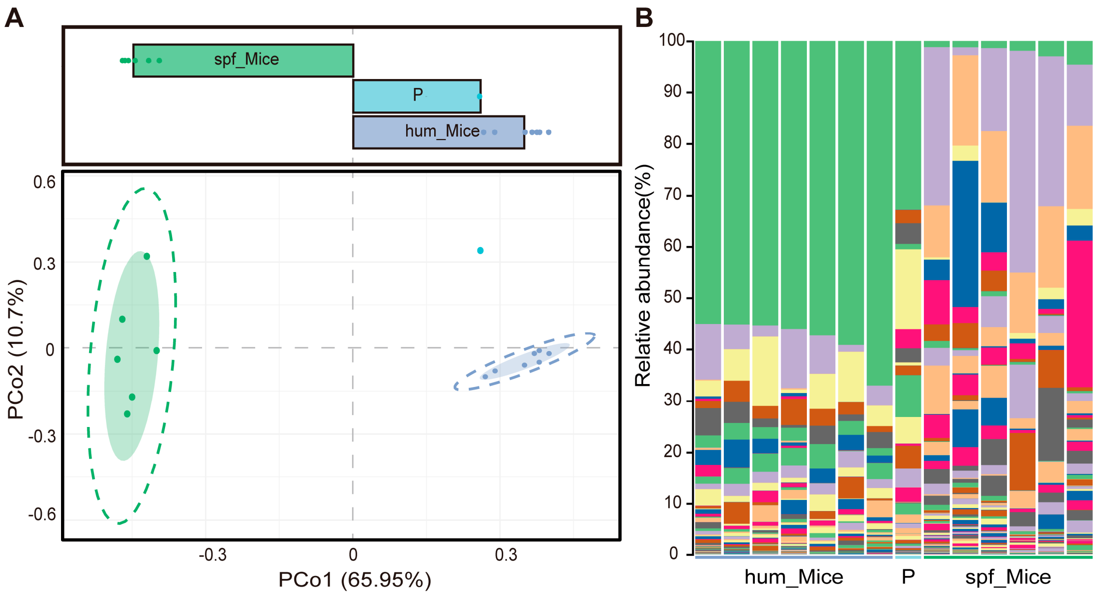

Figure S2 Evaluation of the humanization effect on mice. **A** The Bray-Curtis PCoA plot shows that humanized mice have microbiomes closer to those of the donor. **B** The relative abundance map at the genus level shows that the humanized mouse is very similar to the donor. The colors represent different genera. spf_Mice: unhumanized specific pathogen-free mice; P: humanized fecal donor; hum_Mice: humanized mice

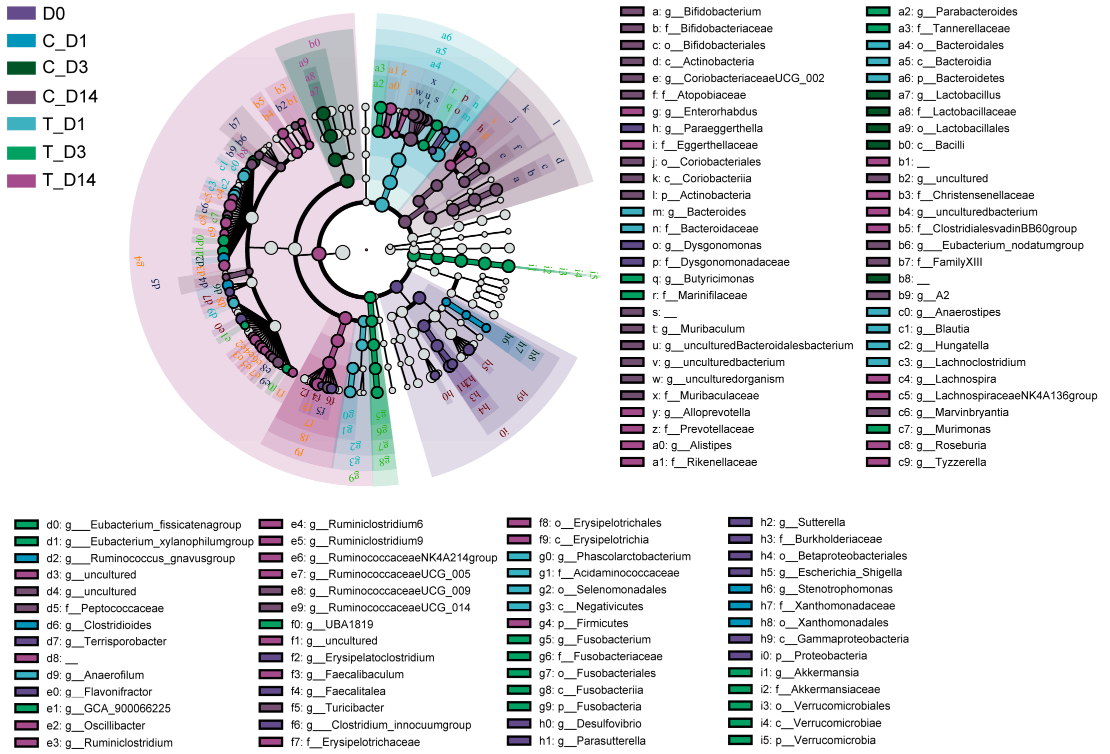

Figure S3 LEfSe analysis was performed on the key bacteria that changed after colon cleansing; the LEfSe cladograms show the results.
